# A new diagnostic algorithm using biopsy specimens in adult T-cell leukemia/lymphoma: combination of RNA in situ hybridization and quantitative PCR for HTLV-1

**DOI:** 10.1038/s41379-020-0635-8

**Published:** 2020-08-17

**Authors:** Mitsuyoshi Takatori, Shugo Sakihama, Megumi Miyara, Naoki Imaizumi, Takashi Miyagi, Kazuiku Ohshiro, Iwao Nakazato, Masaki Hayashi, Junpei Todoroki, Satoko Morishima, Hiroaki Masuzaki, Takuya Fukushima, Kennosuke Karube

**Affiliations:** 1 Department of Pathology and Cell Biology, Graduate School of Medicine and Faculty of Medicine, University of the Ryukyus, Okinawa, Japan; 2 Department of Health and Nutrition, Faculty of Health and Nutrition, Okinawa University, Okinawa, Japan; 3 Laboratory of Molecular Genetics, Graduate School of Health Sciences and Faculty of Medicine, University of the Ryukyus, Okinawa, Japan; 4 Department of Hematology, Heartlife Hospital, Okinawa, Japan; 5 Department of Hematology, Okinawa Prefectural Nanbu Medical Center and Children’s Medical Center, Okinawa, Japan; 6 Department of Pathology, Okinawa Prefectural Nanbu Medical Center and Children’s Medical Center, Okinawa, Japan; 7 Department of Hematology, Nakagami Hospital, Okinawa, Japan; 8 Division of Hematology, Chubu Tokushukai Hospital, Okinawa, Japan; 9 Division of Endocrinology, Diabetes and Metabolism, Hematology, Rheumatology (Second Department of Internal Medicine), Graduate School of Medicine and Faculty of Medicine, University of the Ryukyus, Okinawa, Japan; 10 Laboratory of Hematoimmunology, Graduate School of Health Sciences and Faculty of Medicine, University of the Ryukyus, Okinawa, Japan

**Keywords:** T-cell lymphoma, Tumour virus infections

## Abstract

Histopathological distinction between adult T-cell leukemia/lymphoma (ATLL) and other T-cell neoplasms is often challenging. The current gold standard for the accurate diagnosis of ATLL is the Southern blot hybridization (SBH) assay, which detects clonal integration of human T-cell leukemia virus type I (HTLV-1) provirus. However, SBH cannot be performed with small biopsy or formalin-fixed paraffin-embedded (FFPE) tissue samples because this assay requires a large amount of DNA without degradation. Here we developed a new diagnostic algorithm for the accurate diagnosis of ATLL using FFPE samples. This method combines two HTLV-1 detection assays, namely, ultrasensitive RNA in situ hybridization using RNAscope for *HTLV-1 bZIP factor* (*HBZ*-RNAscope), and quantitative PCR targeting the *tax* gene (*tax*-qPCR). We analyzed 119 FFPE tissue specimens (62 ATLL, and 57 non-ATLL, including 41 HTLV-1 carriers) and compared them with the SBH results using the corresponding fresh-frozen samples. As a result, *tax*-qPCR had a higher ATLL identification rate than *HBZ*-RNAscope (88% [52/59], and 63% [39/62], respectively). However, *HBZ*-RNAscope clearly visualized the localization of HTLV-1-infected tumor cells and its identification rate increased to 94% (17/18) when the analysis was limited to samples up to 2 years old, indicating its usefulness in the daily diagnosis. The diagnostic algorithm combining these two assays successfully evaluated 94% (112/119) of samples and distinguished ATLL from non-ATLL cases including HTLV-1 carriers with 100% sensitivity and specificity. This method is expected to replace SBH and increase the accuracy of the diagnosis of ATLL.

## Introduction

Adult T-cell leukemia/lymphoma (ATLL) is a lymphoid malignancy with a very poor prognosis that is caused by infection with human T-cell leukemia virus type I (HTLV-1) [1, 2]. The development of ATLL is attributed to the effects of viral transcripts, especially Tax [3] and *HTLV-1 bZIP factor* (*HBZ*) [4], as well as the accumulation of somatic genetic abnormalities over a long period of time.

An accurate diagnosis of ATLL requires identifying atypical lymphocytes by cytological or histological methods, and confirming clonal proliferation of HTLV-1-infected cells, usually by Southern blot hybridization (SBH) [5]. As large amounts of DNA (as much as 10–15 µg per sample) are needed for SBH [6], raw or frozen peripheral blood mononuclear cells (PBMCs), or fresh-frozen tissue specimens, are typically used [5]. It is as such impossible to perform SBH with small biopsy or formalin-fixed paraffin-embedded (FFPE) samples, which are commonly used for pathological diagnoses. Furthermore, performing SBH is a complicated procedure and difficult in general hospital laboratories. Consequently, some cases are diagnosed as “tentative” ATLL based on concordant clinical and laboratory data including histopathological assessment, without confirmation by SBH [7, 8]. However, it is possible to overlook peripheral T-cell lymphoma or anaplastic large cell lymphoma, which histologically mimic ATLL, in patients with HTLV-1 infection [7].

The direct detection of viral transcripts in tissue specimens is critical for a definitive diagnosis of virus-associated tumors such as lymphomas positive for Epstein–Barr virus-encoded small RNAs [9], and cervical cancers expressing human papillomavirus E6/E7 mRNA [10]. However, a simple and reliable method for detecting HTLV-1 in tissue is yet to be established [11], mainly because the 5′ end of the HTLV-1 proviral genome harboring the long terminal repeat (LTR) is frequently deleted or methylated in ATLL cells; leading to the repression of some viral transcripts [12–14]. Recent studies have employed RNA in situ hybridization for *HBZ* [15] (located near the 3′ LTR and expressed in all ATLL cells) [16], and quantitative PCR amplification of the *tax* gene (located in the highly conserved *pX* region) using DNA extracted from FFPE tissue samples (*tax*-qPCR) [17]. Although these two methods improve the sensitivity of HTLV-1 detection in FFPE specimens, it remains unclear whether they can clearly distinguish between ATLL and a nonneoplastic HTLV-1 carrier status. In theory, the two methods can identify HTLV-1 in tissue specimens of patients who are carriers and, as such, the detection of viral products does not always reflect malignancy. Moreover, the sensitivity and specificity of these methods and their concordance with SBH are unknown. Therefore, a reliable strategy for the routine histopathological diagnosis of ATLL is needed.

The purpose of this study was to establish a novel diagnostic method using FFPE tissue samples to replace SBH. To this end, we evaluated the effectiveness of ultrasensitive *HBZ* in situ hybridization using RNAscope (*HBZ*-RNAscope) and *tax*-qPCR determining HTLV-1 proviral load (PVL). By combining the two approaches with an appropriate cutoff value, we established an algorithm that successfully distinguishes between ATLL and non-ATLL (carriers or noncarriers) with 100% sensitivity and specificity. Thus, we propose the application of our rapid and accurate SBH-free diagnostic algorithm to the routine pathological diagnosis of ATLL.

## Materials and methods

### Samples

Samples and data were provided from five hospitals in the Okinawa prefecture (Ryukyu University Hospital, Heartlife Hospital, Okinawa Prefectural Nanbu Medical Center and Children’s Medical Center, Nakagami Hospital, and Chubu Tokushukai Hospital), which is an HTLV-1 endemic area. A total of 62 ATLL cases diagnosed between 2010 and 2019 (lymph node [*n* = 55], skin [*n* = 3], subcutaneous mass [*n* = 2], pleural effusion [*n* = 1], and breast [*n* = 1]), in which both SBH results and FFPE tissue specimens were obtained, were selected. In addition, FFPE samples from 41 non-ATLL lesions developed in HTLV-1 carriers (T-cell neoplasm [*n* = 3], non-T-cell neoplasm [*n* = 10], myelodysplastic syndrome [*n* = 1], carcinoma [*n* = 10], sarcoma [*n* = 1], and nonneoplastic lesion [*n* = 16]), and 16 tissue samples from non-HTLV-1 carriers (lymphoid tumor [*n* = 2], carcinoma [*n* = 8], and nonmalignant lesion [*n* = 6]), were collected for comparison with ATLL samples. The three cases with T-cell neoplasms included in this study comprised two cases with angioimmunoblastic T-cell lymphoma (AITL), and one case with extranodal NK/T-cell lymphoma. SBH was performed in one AITL case and showed the absence of monoclonal viral integration. Although the other two cases lacked SBH results, a detailed analysis of clinical, morphological, and immunophenotypical findings excluded the possibility of ATLL [18]. HTLV-1 carrier status was determined based on the detection of serum HTLV-1 antibodies in the peripheral blood by a particle agglutination assay as previously described [19]. The data for all cases is summarized and presented in the Supplementary Table 1. This study was approved by the institutional review boards of the University of the Ryukyus and all participating institutions.

### Ultrasensitive RNA in situ hybridization targeting *HBZ*

**Fig. 1:**
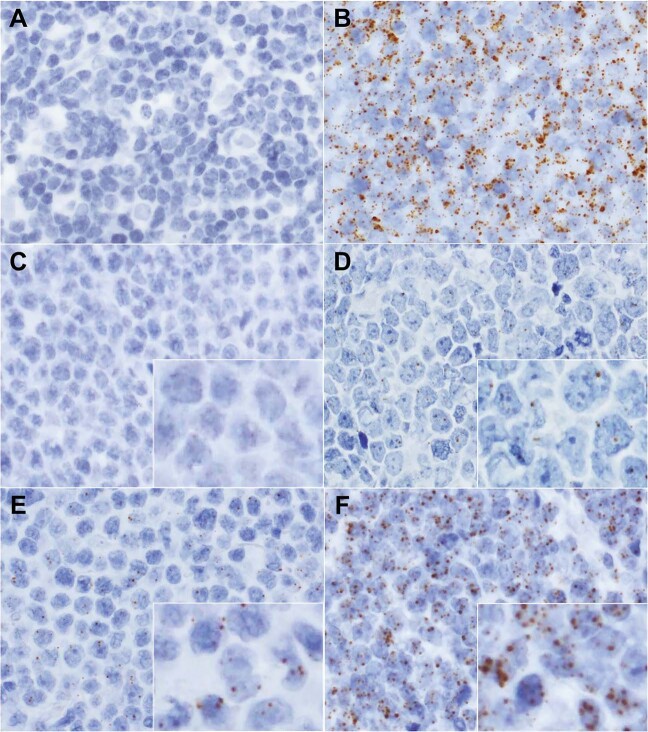
Evaluation of *HBZ*-RNAscope. **a**, **b** *PPIB* signals in representative cases. **a** This case (case no. 2) was regarded as non-evaluable as almost no *PPIB* signal was observed. **b** In this evaluable case (case no. 39), *PPIB* signals were detected in most cells. **c**–**f** *HBZ* signals. **c** This case (case no. 1) showed almost no nuclear *HBZ* signal. Even when some signals were observed, it was difficult to determine whether they were identical to those in tumor cells. Such cases were scored as ±. **d** A case (case no. 35) scored as 1+. A subset of tumor cells showed clear positive signals. **e** A case (case no. 14) scored as 2+. Positive signals were evenly distributed in most tumor cells. **f** A case (case no. 46) scored as 3+. Clusters of positive signals were easily identifiable. Original magnification, ×400 (**a**–**f**). *HBZ*-RNAscope, ultrasensitive RNA in situ hybridization for *HTLV-1 bZIP factor* using RNAscope; *PPIB, peptidylprolyl isomerase B*.

*HBZ*-RNAscope was performed with FFPE samples from 62 ATLL patients, 14 HTLV-1 carriers (T-cell neoplasm [*n* = 3], non-T-cell neoplasm [*n* = 4], and nonneoplastic lesion [*n* = 7]), and 2 non-HTLV-1 carriers (lymphoid tumor [*n* = 1], and nonmalignant lesion [*n* = 1]) using RNAscope (ACDBio, Newark, CA, USA) [20] according to the manufacturer’s protocol. A probe (ACDBio; cat. no. 432901) targeting *HBZ* RNA, which is primarily located in the nucleus [21], was used to detect HTLV-1 (Supplementary Fig. 1). As a positive control, we used a probe targeting the transcript of the housekeeping gene *peptidylprolyl isomerase B* (*PPIB*) (ACDBio; cat. no. 313901). Samples without positive *PPIB* signals were defined as non-evaluable (Fig. 1a, b). For evaluable samples, the *HBZ* signal intensity was scored as ±, 1+, 2+, or 3+ (Fig. 1c–f). When a few signal-positive cells/high-power field were observed but they were difficult to distinguish as tumor cells, we defined the score as ± (Fig. 1c). A score of 1+ was defined as clear signal-positive tumor cells, but with an uneven distribution (Fig. 1d). The score of 2+ was defined when signal was detected in the majority of tumor cells, but cluster formation was not obvious (Fig. 1e). If the signal was dense and strong and formed clusters, a score of 3+ was given (Fig. 1f).

### Quantitative real-time PCR for HTLV-1 PVL

DNA was extracted from all 119 FFPE samples and quantified as previously described [22]. A sufficient amount of DNA was obtained from each sample. As elaborated in the Supplementary Method, TaqMan Fast Advanced Master Mix (Thermo Fisher Scientific, Waltham, MA) was used with 10–30 ng of DNA for quantitative real-time PCR on a StepOnePlus Real-Time PCR System (Thermo Fisher Scientific). A standard curve was generated using serially diluted DNA from TL-Om1 [23], an ATLL cell line; *HBB* (a gene coding for β-globin) was quantified as an internal control. PVL (%) was defined as the number of copies of HTLV-1 provirus per 100 nucleated cells based on the assumption that there are two copies of *HBB* gene per cell. PVL was calculated as an average value of three simultaneous measurements. Primers and probes used in this study were based on a previous study (Supplementary Fig. 1 and Supplementary Table 2) [24].

### SBH and DNA quality analysis

SBH was performed using DNA extracted from raw tissue samples matching the FFPE specimens from 62 ATLL patients and 5 non-ATLL HTLV-1 carriers. The experimental procedure has been previously described [25]. The results of SBH performed in the collaborating institutions were also used. TapeStation (Agilent, Tokyo, Japan) was used to verify DNA quality according to the manufacturer’s protocol.

## Results

### HTLV-1 detection by *HBZ*-RNAscope

**Fig. 2:**
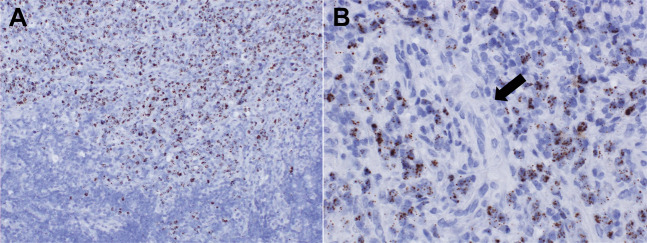
Identification of HTLV-1-infected cells detected by *HBZ*-RNAscope in lymph node. In case no. 54, **a** *HBZ*-positive cells were visible in the upper half of the figure but were mostly absent in the lower half. **b** While many lymphoma cells showed *HBZ* positivity, blood vessels (arrow) were negative. Thus, the extent of ATLL cell infiltration into the tissue was clearly visible. Original magnification, ×200 (**a**), ×400 (**b**). *HBZ*-RNAscope, ultrasensitive RNA in situ hybridization for *HTLV-1 bZIP factor* using RNAscope; HTLV-1, human T-cell leukemia virus type I; ATLL, adult T-cell leukemia/lymphoma.

Of the 62 ATLL cases, 51 (82%) were evaluable based on clear detection of the *PPIB* signal. The *HBZ* signal showed variable intensity depending on the case. In samples that were strongly positive for *HBZ* (2+ and 3+), the border between infiltrating tumor cells and normal tissue was clearly distinguishable (Fig. 2). Samples with an uneven distribution of *HBZ*-positive tumor cells were scored as 1+ (Supplementary Fig. 2). Some artifacts, such as an unusually large (Supplementary Fig. 3A) or faint (Supplementary Fig. 3B) signal, were observed in non-HTLV-1 carrier samples. These signals were often indistinguishable from few positive signals observed in non-ATLL lesions of HTLV-1 carriers. To eliminate the possibility of pseudopositivity and accurately identify ATLL, 12 cases that were *HBZ *± were considered as negative in our analysis. In total, 39 cases of *HBZ* ≥1+ were determined to be positive, corresponding to a diagnosis of ATLL with a sensitivity of 76% (39/51).

### HTLV-1 provirus quantification in FFPE samples

**Fig. 3:**
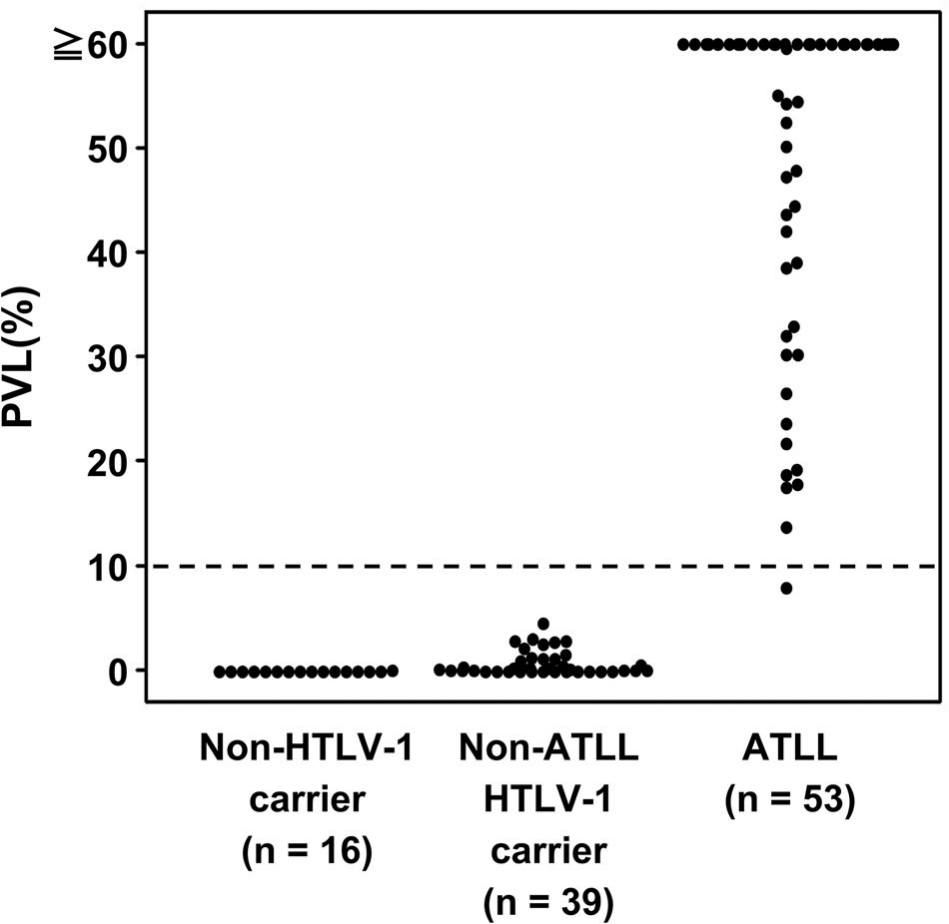
HTLV-1 PVL in each group and cutoff value. PVL of 108 evaluable cases is shown. The broken line indicates the cutoff value of 10% for distinguishing ATLL from other lesions (i.e., non-ATLL lesions arising in HTLV-1 carriers and non-HTLV-1 carriers). HTLV-1, human T-cell leukemia virus type I; PVL, proviral load; ATLL, adult T-cell leukemia/lymphoma.

The assay for HTLV-1 provirus quantification in the previous study was established as an experimental system for PBMCs or fresh-frozen tissue samples, and its application for FFPE samples was not evaluated [24]. We initially performed the assay with both FFPE and matched raw tissue samples for the purpose of comparison and found a high degree of correlation between the sample types (coefficient of determination [*R*^2^] = 0.845, *P* < 0.001) (Supplementary Fig. 4), implying that FFPE samples could be used for the assay. However, the accuracy of PVL was low in samples with an extremely low copy number of *HBB* (Supplementary Fig. 5). Therefore, only cases with an *HBB* copy number ≥200 were considered as evaluable. According to this criterion, 110/119 (92%) cases were regarded to be evaluable. The PVL of 39 non-ATLL tissue samples from HTLV-1 carriers ranged from 0 to 4.6% with a mean value of 0.8% (Fig. 3). Even when limited to the 14 samples of reactive lesions, the mean PVL value was 1.4% (range: 0–4.6%). The value 4 standard deviations away from the mean was 5.4%. Based on these findings, we set the cutoff value as 10%. In light of this cutoff value, it did not seem appropriate to include cases with low amounts of tumor and we excluded three cases with tumor ratios <30% (case no. 16, 32, and 54). One of these did in fact show a low PVL below the cutoff value. Thus, a total of 116 cases were analyzed. This cutoff value evaluated as positive for 52 out of 53 ATLLs (range: 8.0–170.8%, mean value: 66.0%) and negative for all non-ATLLs (HTLV-1 carrier [*n* = 39], non-HTLV-1 carrier [*n* = 16]) among 108 evaluable cases (Fig. 3). Therefore, this assay distinguished ATLL from non-ATLL, irrespective of HTLV-1 carrier status, with 98% (52/53) sensitivity and 100% (55/55) specificity.

### Comparison with the SBH assay

**Fig. 4:**
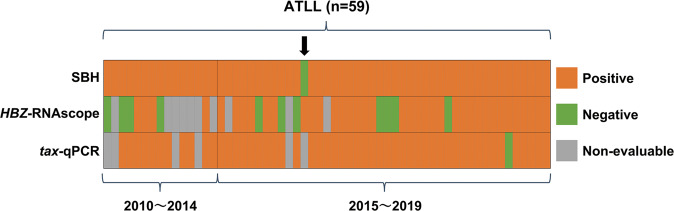
Comparisons between results obtained by SBH, *HBZ*-RNAscope, and *tax*-qPCR in ATLL cases. Columns depict individual cases and rows show the results of SBH, *HBZ*-RNAscope, and *tax*-qPCR. Results from each analysis are indicated by color (orange = positive, green = negative, gray = non-evaluable) and are ordered by year of biopsy and divided into two groups (biopsied in 2010–2014 and 2015–2019). The arrow indicates a case in which monoclonal integration of HTLV-1 was not detected by SBH, but that was ultimately diagnosed as ATLL based on clinical, histopathological, and laboratory findings. SBH, southern blot hybridization; *HBZ*-RNAscope, ultrasensitive RNA in situ hybridization for *HTLV-1 bZIP factor* using RNAscope; *tax*-qPCR, quantitative PCR detection of the *tax* gene; ATLL, adult T-cell leukemia/lymphoma.

We compared results for the 59 ATLL cases with a tumor ratio of 30% or more in the specimens, and in which SBH, *HBZ*-RNAscope, and *tax*-qPCR were performed (Fig. 4). Of the 58 cases in which monoclonal integration of HTLV-1 provirus was confirmed by SBH, 37 were identified as positive by *HBZ*-RNAscope and 52 by *tax*-qPCR. One case with a negative SBH result was ultimately diagnosed as ATLL based on a combination of the other clinicopathological findings (Fig. 4 and Supplementary Fig. 6A). This sample was biopsied from a case that showed lymph node re-enlargement 2 months after bone marrow transplantation following a primary diagnosis of ATLL. SBH with PBMCs as the primary lesion showed clear monoclonal integration of HTLV-1 (data not shown). The histopathological diagnosis of the relapsed lesion was peripheral T-cell lymphoma, with >90% of the tumor cells positive for cluster of differentiation (CD)3, CD4, CD8, and CD25 CC chemokine receptor 4 by immunohistochemistry. Based on this result and clinical symptoms, the case was diagnosed as recurrent ATLL and treatment was initiated before SBH results, which were returned to the hematologist. Although this sample was non-evaluable by *tax*-qPCR, clear positive staining was observed by *HBZ*-RNAscope using a FFPE sample (Supplementary Fig. 6B). Given the clinical course and *HBZ*-RNAscope positivity, a false-negative SBH result was suspected and further analysis was carried out. Clonal rearrangement of the T-cell receptor gene was confirmed by PCR following the BIOMED-2 protocol (Supplementary Fig. 6C) [26]. The PVL of the fresh tissue sample used in the SBH analysis was extremely high (217%). The DNA integrity number of this case was 4.6, indicating extremely poor DNA quality compared with the scores of 8.0 and 8.5 for the two samples in which monoclonal integration of HTLV-1 was clearly detected by SBH (Supplementary Fig. 6D). We therefore concluded that this case was found to be falsely negative by SBH due to poor DNA quality. On the other hand, the other five cases that showed a negative result by SBH were also negative by *HBZ*-RNAscope and *tax*-qPCR.

To evaluate the influence of nucleic acid degradation in FFPE samples on ATLL identification, cases in which biopsy was performed >5 years ago (old cases) and within the previous 5 years (recent cases) were separately analyzed (Fig. 4). In the older cases, the ATLL detection rate was 27% (4/15) by *HBZ*-RNAscope and 73% (11/15) by *tax*-qPCR; indicating the latter had higher detection power, even with degraded DNA (*P* = 0.027, Fisher’s exact test). Seven of the fifteen older cases (47%) were non-evaluable by *HBZ*-RNAscope due to *PPIB* negativity, and the remaining four cases (27%) were regarded as falsely negative based on the discrepancy in results obtained by *HBZ*-RNAscope (negative) and SBH (positive). For the more recent cases (*n* = 44), *HBZ*-RNAscope showed a higher detection rate of 77% (34/44), and a lower false-negative rate of 16% (7/44), whereas *tax*-qPCR identified ATLL in 93% (41/44) of the recent cases. Notably, in 94% (16/17) of the most recent cases (<2 years of storage), the results of *HBZ*-RNAscope were consistent with those obtained by SBH. Furthermore, 71% (12/17) of the samples stored for <2 years showed *HBZ* scores of 2+ or 3+, compared with 20% (3/15) of those cases with samples stored for >5 years, indicating that the intensity of *HBZ*-RNAscope was affected by age-related degradation of nucleic acids.

### Proposal of a novel diagnostic algorithm

**Fig. 5:**
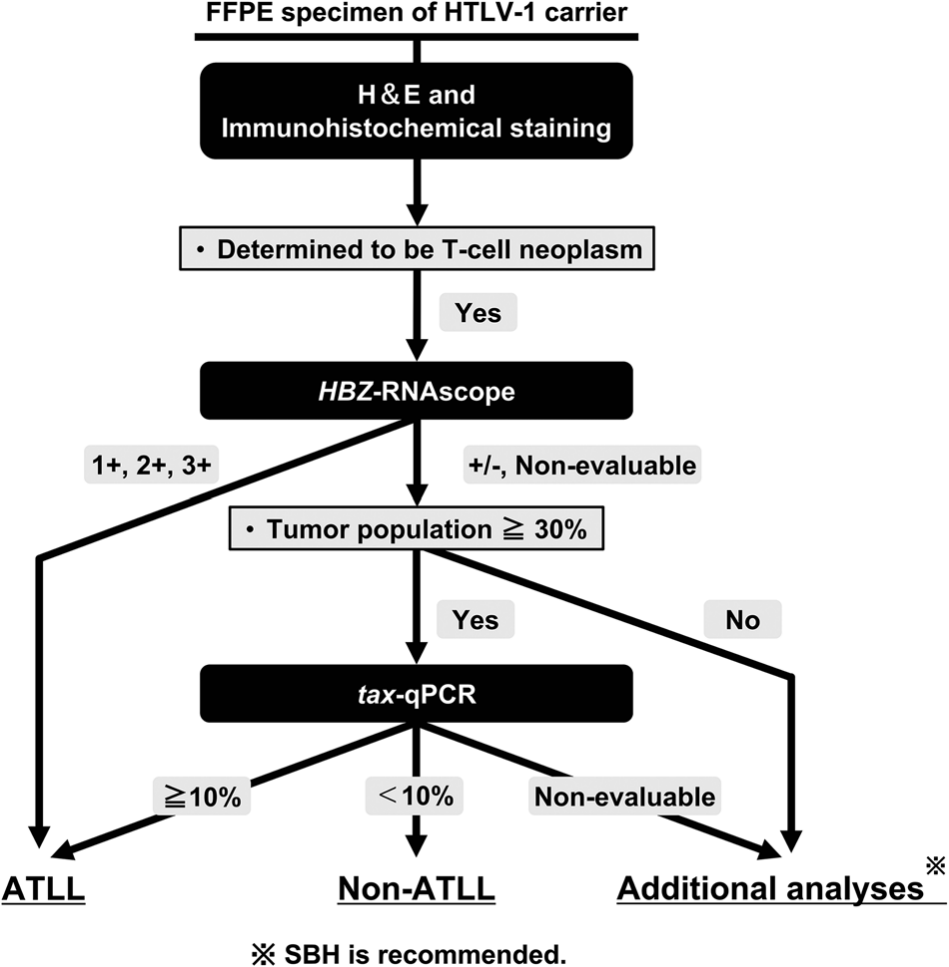
Novel algorithm for histological diagnosis of ATLL. FFPE, formalin-fixed paraffin-embedded; HTLV-1, human T-cell leukemia virus type I; H&E, hematoxylin and eosin; *HBZ*-RNAscope, ultrasensitive RNA in situ hybridization for *HTLV-1 bZIP factor* using RNAscope; *tax*-qPCR, quantitative PCR detection of the *tax* gene; ATLL, adult T-cell leukemia/lymphoma; SBH, southern blot hybridization.

Based on the results of this study, we propose a novel diagnostic algorithm for ATLL (Fig. 5). According to the algorithm, 94% (112/119) of samples were evaluable with 100% sensitivity and specificity. Furthermore, the algorithm was able to identify an ATLL case that was falsely negative by SBH, indicating a high degree of accuracy and reliability.

## Discussion

Histological analysis alone cannot distinguish ATLL from non-ATLL T-cell tumors in HTLV-1 carriers [7], and there is a need for a practical ATLL diagnostic method, especially in cases in which SBH cannot be performed. In this study, we carried out *HBZ*-RNAscope and quantified HTLV-1 PVL using FFPE samples from ATLL patients and non-ATLL HTLV-1 carriers, and established a diagnostic algorithm applicable to FFPE samples with 100% sensitivity and specificity (Fig. 5).

In the application of *tax*-qPCR to FFPE samples, we set a cutoff value for *HBB* gene as an internal control, which was not used in the previous study with fresh PBMCs [24]. This eliminated the potential effects of nucleic acid degradation in FFPE samples due to various factors such as time between surgery and formalin fixation, type of formalin, time of fixation, and storage period [27, 28]; this is in contrast to nucleic acids derived from raw or frozen specimens, which are usually of sufficient quality for PCR analysis. Of the FFPE samples that had been stored for >5 years, 27% were evaluable by *HBZ*-RNAscope and 73% by *tax*-qPCR (Fig. 4), indicating that the latter method is superior for analyzing older samples and is thus more appropriate for retrospective studies.

Although *tax*-qPCR alone has 98% sensitivity and 100% specificity, the proposed algorithm recommends performing *HBZ*-RNAscope first. This is mainly because *HBZ*-RNAscope renders the viral product in tumor cells visible and reveals the extent of tumor cell infiltration into tissues (Fig. 2). In the present study, the detection rate of ATLL by *HBZ*-RNAscope was 94% in samples that had been stored for <2 years; this suggests that this assay is suitable for routine diagnostic testing, which is usually performed on samples immediately after biopsy or surgery. In addition, positive signals were obtained by *HBZ*-RNAscope in two ATLL cases that were non-evaluable and false-negative by *tax*-qPCR, further underscoring its utility as the first screening test. A further reason is that the *HBZ*-RNAscope procedure is relatively simple as the test material is mounted on a glass slide and the assay can be performed with an automated protocol in the general pathology laboratory of a hospital [29]. In terms of turnaround time, the experimental procedure can be completed in 1 day, allowing the quick return of diagnostic test results to clinicians [20]. Unlike SBH, *HBZ*-RNAscope and *tax*-qPCR do not reveal the clonality of tumor cells. Although clonal proliferation can be confirmed by PCR analysis of T-cell receptor gene rearrangement, the proposed algorithm does not require detecting the clonality of HTLV-1-infected cells because it is applied only to samples in which tumor cells are histologically identifiable, thereby confirming a neoplastic state.

This study included an ATLL case that was negative by SBH as a result of DNA degradation in the fresh tissue specimen. DNA used for SBH is presumed to be of high quality as it is usually extracted from PBMCs or fresh-frozen tissue. Therefore, detailed confirmation of DNA quality is not often performed. There have been four case reports of non-ATLL T-cell tumors in HTLV-1 carriers [30–33]; our findings suggest that eliminating the possibility of false negatives is important for such cases, and this can be achieved using the proposed algorithm in this study.

Given that all experiments in this study were performed at a single laboratory, interlaboratory reproducibility must be evaluated in the future. The reproducibility of *tax*-qPCR seems high because a standardized experimental method using TL-Om1, used in the present study, has already been established in the assay of PBMCs [34, 35]. Although the sensitivity of *HBZ*-RNAscope may vary across facilities, as this technique is vulnerable to nucleic acid degradation that can occur during long storage periods, the effect could be minimized when the analysis is limited to fresh FFPE samples in daily pathological diagnoses.

Distinguishing ATLL from non-ATLL T-cell tumors arising in HTLV-1 carriers is critical but often cannot be achieved based solely on histological findings [7]. In the present study, *HBZ*-RNAscope was performed on seven non-ATLL lymphoid tumors, including three T-cell tumors that arose in HTLV-1 carriers. While the tumor cells were negative, a small number of scattered *HBZ*-positive cells were observed (Supplementary Fig. 7). These few signals, which were scored as ± in the present algorithm, could correspond to nonneoplastic HTLV-1-infected T cells, or may be artifacts such as those observed in non-HTLV-1 carrier samples (Supplementary Fig. 3). *HBZ*-RNAscope cannot be used to determine whether the patient is an HTLV-1 carrier, which should be based on conventional serological testing. Furthermore, our algorithm is not applicable to cases in which tumor cells cannot be clearly distinguished from reactive atypical cells, as is sometimes observed in skin or lymph node lesions. On the other hand, as shown in Supplementary Fig. 8, *HBZ*-RNAscope can be evaluable for cases in which tumor cell aggregation is visible, even if the proportion of tumor cells in the whole tissue is low.

This study mainly included ATLL cases with SBH data and available tissue specimens from 2010 to 2019 at five collaborative institutions. Obtaining a comprehensive investigation of the detailed incidence of ATLL was not possible from all the institutions. However, we obtained comprehensive data from one facility (Ryukyu University Hospital) between 2015 and 2017. From this institution, there were 24 tissue specimens in which the pathological findings were consistent with ATLL, and from which SBH was performed in nine cases. SBH was not performed in the other 15 tissue specimens mainly due to small sample size, and HTLV-1 viral integration was proved only in the peripheral blood of eight of these 15 cases. Considering the above, the proposed algorithm allows more accurate confirmation of ATLL diagnosis in more than half of the biopsied tissue specimens in daily diagnosis.

In conclusion, we propose a novel, rapid, and accurate algorithm using FFPE samples for the histopathological diagnosis of ATLL to replace the SBH assay, which has thus far been the gold standard. Visualizing the location of HTLV-1-infected tumor cells by *HBZ*-RNAscope and quantifying the number of HTLV-1-infected cells by *tax*-qPCR allows ATLL to be distinguished from non-ATLL with a high degree of accuracy (100% sensitivity and specificity). At present, evaluating samples that are borderline between tumoral and reactive, remains challenging, even with this new algorithm. Further studies are needed to determine whether it can be applied successfully to these challenging lesions.

## Supplementary information

Supplementary Information
