# Supplementary material for: A new diagnostic algorithm using biopsy specimens in adult T-cell leukemia/lymphoma: combination of RNA in situ hybridization and quantitative PCR for HTLV-1
Source: Mod Pathol. 2020 Aug 17;34(1):51–8. doi: 10.1038/s41379-020-0635-8 (PMC7806504; doi:10.1038/s41379-020-0635-8)
Supplement: Supplementary file 1 — Supplementary Information [file 41379_2020_635_MOESM1_ESM.pdf]

Supplementary Table

Supplementary Table 1. Sample information and results of analyses.

Case no.	Year of collection	Specimen	Diagnosis	Anti-HTLV-1 antibody	Southern blot hybridization	ATLL tumor ratio (%)	*HBZ*-RNAscope	*tax*-qPCR (%)
1	2010	Lymph node	Adult T-cell leukemia/lymphoma	Positive	Positive	80	±	n.e.
2	2010	Lymph node	Adult T-cell leukemia/lymphoma	Positive	Positive	90	n.e.	n.e.
3	2011	Lymph node	Adult T-cell leukemia/lymphoma	Positive	Positive	90	±	17.9
4	2011	Lymph node	Adult T-cell leukemia/lymphoma	Positive	Positive	30	±	21.8
5	2013	Lymph node	Adult T-cell leukemia/lymphoma	Positive	Positive	70	2+	26.6
6	2013	Lymph node	Adult T-cell leukemia/lymphoma	Positive	Positive	>95	2+	132.1
7	2013	Lymph node	Adult T-cell leukemia/lymphoma	Positive	Positive	90	1+	60.0
8	2013	Lymph node	Adult T-cell leukemia/lymphoma	Positive	Positive	90	±	54.5
9	2013	Lymph node	Adult T-cell leukemia/lymphoma	Positive	Positive	90	n.e.	17.6
10	2013	Lymph node	Adult T-cell leukemia/lymphoma	Positive	Positive	70	n.e.	n.e.
11	2013	Lymph node	Adult T-cell leukemia/lymphoma	Positive	Positive	90	n.e.	30.3
12	2014	Skin	Adult T-cell leukemia/lymphoma	Positive	Positive	80	n.e.	33.0
13	2014	Skin	Adult T-cell leukemia/lymphoma	Positive	Positive	80	n.e.	n.e.
14	2014	Lymph node	Adult T-cell leukemia/lymphoma	Positive	Positive	90	2+	89.5
15	2014	Lymph node	Adult T-cell leukemia/lymphoma	Positive	Positive	50	n.e.	105.5
16	2014	Pleural effusion	Adult T-cell leukemia/lymphoma	Positive	Positive	20	n.e.	n.e.
17	2015	Lymph node	Adult T-cell leukemia/lymphoma	Positive	Positive	90	2+	39.1
18	2015	Subcutaneous mass	Adult T-cell leukemia/lymphoma	Positive	Positive	90	n.e.	18.8
19	2015	Skin	Adult T-cell leukemia/lymphoma	Positive	Positive	80	2+	87.3
20	2015	Lymph node	Adult T-cell leukemia/lymphoma	Positive	Positive	80	2+	23.7
21	2015	Lymph node	Adult T-cell leukemia/lymphoma	Positive	Positive	90	1+	170.8
22	2015	Lymph node	Adult T-cell leukemia/lymphoma	Positive	Positive	>95	±	115.3
23	2015	Lymph node	Adult T-cell leukemia/lymphoma	Positive	Positive	80	2+	63.7
24	2015	Lymph node	Adult T-cell leukemia/lymphoma	Positive	Positive	70	2+	47.3
25	2015	Lymph node	Adult T-cell leukemia/lymphoma	Positive	Positive	50	±	38.6
26	2015	Subcutaneous mass	Adult T-cell leukemia/lymphoma	Positive	Positive	80	n.e.	n.e.
27	2015	Lymph node	Adult T-cell leukemia/lymphoma	Positive	Positive	90	±	55.1
28	2016	Lymph node	Adult T-cell leukemia/lymphoma	Positive	Negative	90	1+	n.e.
29	2016	Lymph node	Adult T-cell leukemia/lymphoma	Positive	Positive	40	1+	13.8
30	2016	Lymph node	Adult T-cell leukemia/lymphoma	Positive	Positive	90	1+	90.7
31	2016	Lymph node	Adult T-cell leukemia/lymphoma	Positive	Positive	30	n.e.	19.3
32	2016	Lymph node	Adult T-cell leukemia/lymphoma	Positive	Positive	5	±	20.5
33	2016	Lymph node	Adult T-cell leukemia/lymphoma	Positive	Positive	>95	3+	54.3

34	2016	Lymph node	Adult T-cell leukemia/lymphoma	Positive	Positive	70	3+	61.6
35	2016	Lymph node	Adult T-cell leukemia/lymphoma	Positive	Positive	80	1+	47.9
36	2017	Lymph node	Adult T-cell leukemia/lymphoma	Positive	Positive	70	2+	32.1
37	2017	Lymph node	Adult T-cell leukemia/lymphoma	Positive	Positive	90	3+	44.5
38	2017	Lymph node	Adult T-cell leukemia/lymphoma	Positive	Positive	90	3+	139.5
39	2017	Lymph node	Adult T-cell leukemia/lymphoma	Positive	Positive	90	±	98.7
40	2017	Breast	Adult T-cell leukemia/lymphoma	Positive	Positive	90	±	83.7
41	2017	Lymph node	Adult T-cell leukemia/lymphoma	Positive	Positive	90	±	90.1
42	2017	Lymph node	Adult T-cell leukemia/lymphoma	Positive	Positive	50	2+	94.8
43	2017	Lymph node	Adult T-cell leukemia/lymphoma	Positive	Positive	90	2+	42.1
44	2017	Lymph node	Adult T-cell leukemia/lymphoma	Positive	Positive	80	2+	69.3
45	2018	Lymph node	Adult T-cell leukemia/lymphoma	Positive	Positive	90	3+	108.0
46	2018	Lymph node	Adult T-cell leukemia/lymphoma	Positive	Positive	90	3+	155.5
47	2018	Lymph node	Adult T-cell leukemia/lymphoma	Positive	Positive	>95	3+	99.5
48	2018	Lymph node	Adult T-cell leukemia/lymphoma	Positive	Positive	90	±	52.5
49	2018	Lymph node	Adult T-cell leukemia/lymphoma	Positive	Positive	90	1+	59.6
50	2018	Lymph node	Adult T-cell leukemia/lymphoma	Positive	Positive	80	3+	60.3
51	2018	Lymph node	Adult T-cell leukemia/lymphoma	Positive	Positive	90	3+	128.0
52	2018	Lymph node	Adult T-cell leukemia/lymphoma	Positive	Positive	90	2+	43.7
53	2018	Lymph node	Adult T-cell leukemia/lymphoma	Positive	Positive	80	3+	72.7
54	2018	Lymph node	Adult T-cell leukemia/lymphoma	Positive	Positive	20	3+	4.5
55	2019	Lymph node	Adult T-cell leukemia/lymphoma	Positive	Positive	90	3+	82.9
56	2019	Lymph node	Adult T-cell leukemia/lymphoma	Positive	Positive	90	3+	50.2
57	2019	Lymph node	Adult T-cell leukemia/lymphoma	Positive	Positive	80	1+	8.0
58	2019	Lymph node	Adult T-cell leukemia/lymphoma	Positive	Positive	70	1+	63.7
59	2019	Lymph node	Adult T-cell leukemia/lymphoma	Positive	Positive	90	2+	74.9
60	2019	Lymph node	Adult T-cell leukemia/lymphoma	Positive	Positive	80	2+	64.5
61	2019	Lymph node	Adult T-cell leukemia/lymphoma	Positive	Positive	80	1+	30.3
62	2019	Lymph node	Adult T-cell leukemia/lymphoma	Positive	Positive	>95	3+	112.9
63	2015	Lymph node	Reactive lymphadenitis	Positive	Negative	-	±	1.0
64	2016	Lymph node	Reactive lymphadenitis	Positive	Negative	-	±	0.1
65	2016	Lymph node	Angioimmunoblastic T-cell lymphoma	Positive	Negative	-	±	1.6
66	2017	Lymph node	Methotrexate-related lymphoproliferative disorders	Positive	Negative	-	±	0.0
67	2017	Lymph node	Reactive lymphadenitis	Positive	Negative	-	±	3.1
68	2012	Lymph node	Dermatopathic lymphadenopathy	Positive	n.t.	-	±	0.3
69	2013	Lymph node	Diffuse large B-cell lymphoma	Positive	n.t.	-	n.t.	0.0

70	2013	Maxillary antrum	Diffuse large B-cell lymphoma	Positive	n.t.	-	n.t.	n.e.
71	2014	Lymph node	Diffuse large B-cell lymphoma	Positive	n.t.	-	n.t.	n.e.
72	2016	Lymph node	Diffuse large B-cell lymphoma	Positive	n.t.	-	n.t.	0.3
73	2016	Adrenal cortex	Diffuse large B-cell lymphoma	Positive	n.t.	-	n.t.	1.3
74	2017	Lymph node	Follicular lymphoma	Positive	n.t.	-	±	0.0
75	2017	Lymph node	Diffuse large B-cell lymphoma	Positive	n.t.	-	n.t.	0.1
76	2018	Lymph node	Dermatopathic lymphadenopathy	Positive	n.t.	-	±	2.6
77	2018	Lymph node	Dermatopathic lymphadenopathy	Positive	n.t.	-	±	4.6
78	2018	Lymph node	Reactive lymphadenitis	Positive	n.t.	-	±	0.6
79	2018	Oropharynx	Diffuse large B-cell lymphoma	Positive	n.t.	-	±	0.0
80	2018	Bone marrow	Normoplastic bone marrow	Positive	n.t.	-	n.t.	0.0
81	2018	Lymph node	Carcinoma	Positive	n.t.	-	n.t.	0.0
82	2018	Lymph node	Reactive lymphadenitis	Positive	n.t.	-	n.t.	0.0
83	2018	Lymph node	Reactive lymphadenitis	Positive	n.t.	-	n.t.	0.2
84	2018	Lymph node	Carcinoma	Positive	n.t.	-	n.t.	0.1
85	2018	Skin	Kaposi's sarcoma	Positive	n.t.	-	n.t.	0.3
86	2018	Lymph node	Carcinoma	Positive	n.t.	-	n.t.	0.0
87	2018	Lymph node	Dermatopathic lymphadenopathy	Positive	n.t.	-	n.t.	2.9
88	2018	Lymph node	Carcinoma	Positive	n.t.	-	n.t.	0.4
89	2018	Bone marrow	Myelodysplastic syndrome	Positive	n.t.	-	n.t.	0.2
90	2018	Lymph node	Carcinoma	Positive	n.t.	-	n.t.	0.1
91	2018	Lymph node	Reactive lymphadenitis	Positive	n.t.	-	n.t.	0.0
92	2018	Lymph node	Carcinoma	Positive	n.t.	-	n.t.	0.0
93	2018	Lymph node	Reactive lymphadenitis	Positive	n.t.	-	n.t.	0.0
94	2019	Lymph node	Iatrogenic immunodeficiency-associated lymphoproliferative disorder	Positive	n.t.	-	±	0.1
95	2019	Skin	Carcinoma	Positive	n.t.	-	n.t.	0.0
96	2019	Lymph node	Reactive lymphadenitis	Positive	n.t.	-	n.t.	2.9
97	2019	Large intestine	Juvenile polyp	Positive	n.t.	-	n.t.	2.8
98	2019	Lymph node	Carcinoma	Positive	n.t.	-	n.t.	2.2
99	2019	Lymph node	Reactive lymphadenitis	Positive	n.t.	-	n.t.	1.2
100	2019	Tongue	Carcinoma	Positive	n.t.	-	n.t.	1.2
101	2019	Lymph node	Carcinoma	Positive	n.t.	-	n.t.	0.5
102	2019	Subcutaneous mass	Extranodal NK/T cell lymphoma	Positive	n.t.	-	±	0.7
103	2019	Lymph node	Angioimmunoblastic T-cell lymphoma	Positive	n.t.	-	±	0.1
104	2015	Lymph node	Diffuse large B-cell lymphoma	Negative	n.t.	-	n.t.	0.0
105	2018	Lymph node	Carcinoma	Negative	n.t.	-	n.t.	0.1

106	2018	Lymph node	Carcinoma	Negative	n.t.	-	n.t.	0.0
107	2018	Lymph node	Carcinoma	Negative	n.t.	-	n.t.	0.0
108	2018	Lymph node	Reactive lymphadenitis	Negative	n.t.	-	n.t.	0.0
109	2018	Skin	Carcinoma	Negative	n.t.	-	n.t.	0.0
110	2018	Lymph node	Reactive follicular hyperplasia	Negative	n.t.	-	±	0.0
111	2018	Lymph node	Carcinoma	Negative	n.t.	-	n.t.	0.0
112	2018	Lymph node	Carcinoma	Negative	n.t.	-	n.t.	0.0
113	2018	Lymph node	Reactive lymphadenitis	Negative	n.t.	-	n.t.	0.0
114	2018	Subcutaneous mass	Dermoid cyst	Negative	n.t.	-	n.t.	0.0
115	2019	Lymph node	Carcinoma	Negative	n.t.	-	n.t.	0.0
116	2019	Skin	Scar tissue	Negative	n.t.	-	n.t.	0.0
117	2019	Lymph node	Follicular lymphoma and diffuse large B-cell lymphoma	Negative	n.t.	-	±	0.0
118	2019	Lymph node	Carcinoma	Negative	n.t.	-	n.t.	0.0
119	2019	Lymph node	Reactive lymphadenitis	Negative	n.t.	-	n.t.	0.0
120	2019	Skin	Adult T-cell leukemia/lymphoma	Positive	n.t.	20	2+	19.2

HTLV-1, human T-cell leukemia virus type I; ATLL, adult T-cell leukemia/lymphoma; *HBZ*-RNAscope, ultrasensitive RNA *in situ* hybridization for *HTLV-1 bZIP factor* using RNAscope; *tax*-qPCR, quantitative PCR detection of the *tax* gene; n.t., not-tested; n.e., non-evaluable.

Supplementary Method

Experimental procedure for *tax*-qPCR.

To quantify the copy number of the targeted HTLV-1 *tax* gene and *HBB* gene, the TaqMan probe assay was performed. The primers and probes used are listed in Supplementary Table 2 below. The PCR program followed the product protocol, i.e., pre-incubation at 95°C for 20 seconds, followed by a total of 50 cycles at 95°C for 1 second, and 60°C for 20 seconds. The standard curves for both HTLV-1 *tax* and *HBB* gene were generated by diluting DNA extracted from the ATLL cell line, TL-Om1.

Supplementary Table 2. Primers and probes used for *tax*-qPCR targeting the HTLV-1 *tax* gene and *HBB* gene.

HTLV-1 *tax* gene (137 base pair)	
Sense	5'-CCCACTTCCCAGGGTTTGGA-3'
Anti-sense	5'-GGCCAGTAGGGCGTGA-3'
Probe	5'-FAM-CCAGTCTACGTGTTTGGAGCTGTGTACA-TAMRA-3'
***HBB* gene (102 base pair)**	
Sense	5'-GTGCACCTGACTCCTGAGGAGA-3'
Anti-sense	5'-CCTTGATACCAACCTGCCCAG-3'
Probe	5'-FAM-AAGGTGAACGTGGATGAAGTTGGTGG-TAMRA-3'

Supplementary Figure

Supplementary Figure 1. Targets of *HBZ*-RNAscope and *tax*-qPCR in HTLV-1 provirus.

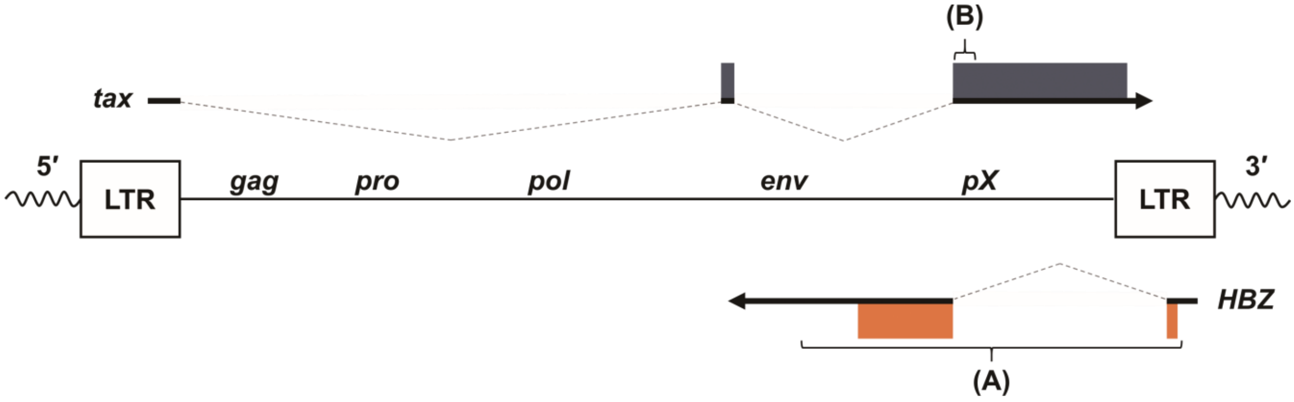

(A) Range covered by the *HBZ*-RNAscope probe. (B) Region corresponding to the amplicon obtained by *tax*-qPCR.

HBZ-RNAscope, ultrasensitive RNA *in situ* hybridization for *HTLV-1 bZIP factor* using RNAscope; *tax*-qPCR, quantitative PCR detection of the *tax* gene; HTLV-1, human T-cell leukemia virus type I.

Supplementary Figure 2. A case with unevenly distributed *HBZ* signals.

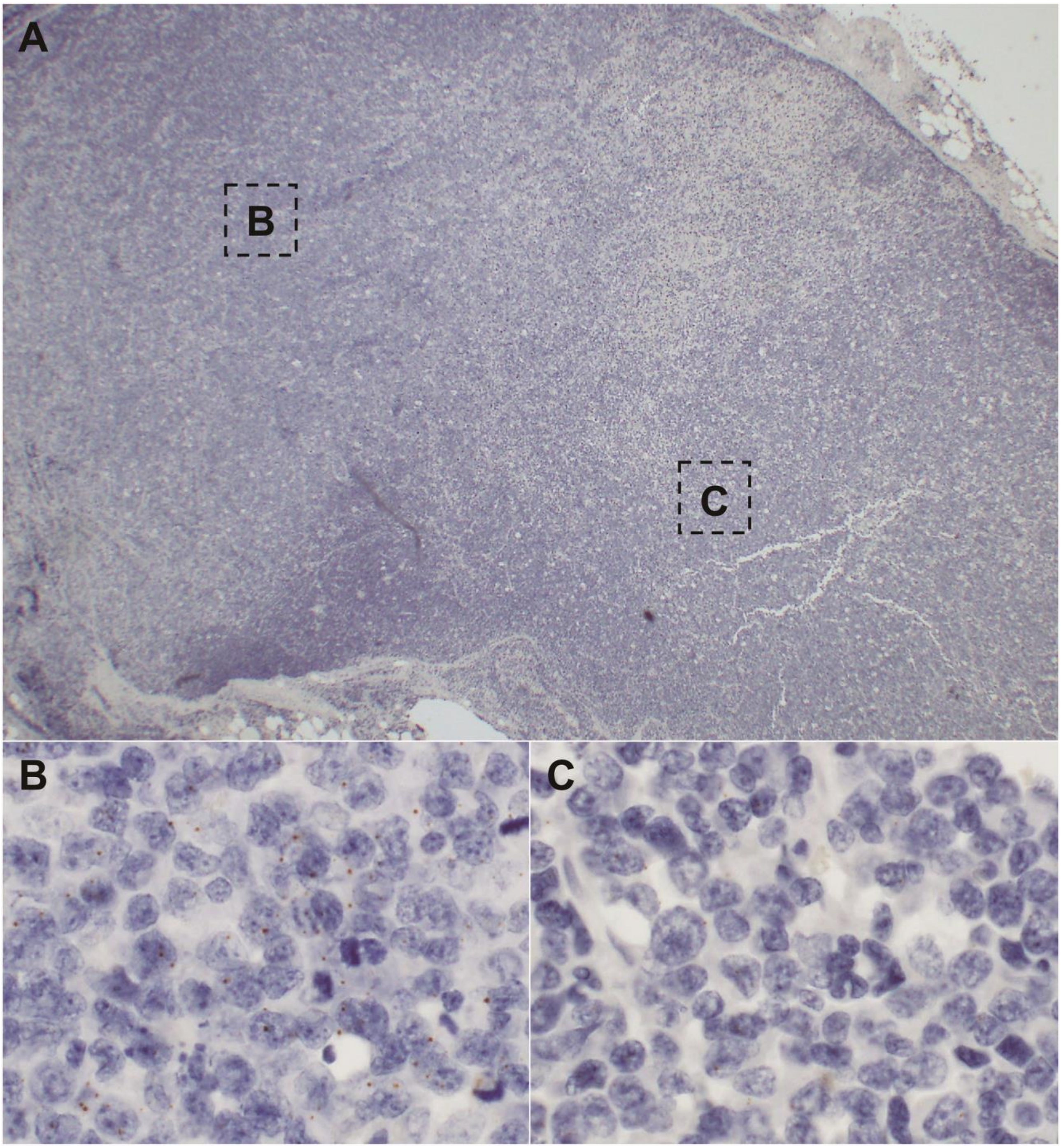

(A-C) *HBZ* signals. (A) A case (case no. 30) of *HBZ*-RNAscope 1+ with uneven signal intensity. (B) Most of the tumor cells were *HBZ*-positive in the field delineated by the broken line. (C) Almost no positive signals were detected in the field. Original magnification, 20× (A), 400× (B, C).

HBZ-RNAscope, ultrasensitive RNA *in situ* hybridization for *HTLV-1 bZIP factor* using RNAscope; HTLV-1, human T-cell leukemia virus type I.

Supplementary Figure 3. Artifacts in *HBZ*-RNAscope identified in non-HTLV-1 carriers.

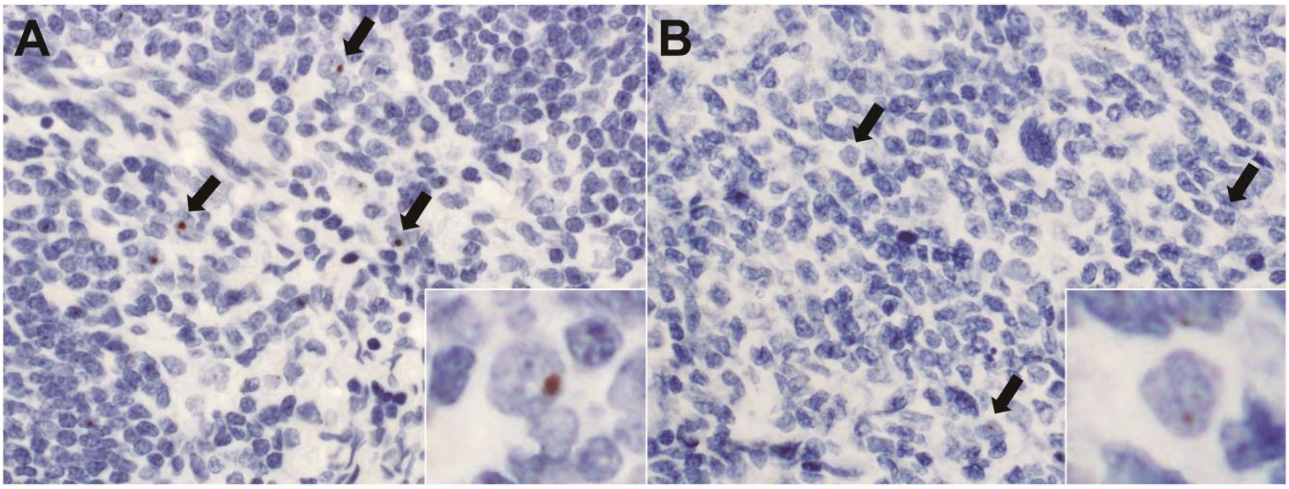

(A) Unusually large blot-like signals were observed in non-HTLV-1 carriers (case no. 110). (B) Several faint signals were also detected (case no. 117). Original magnification, 400× (A, B).

HBZ-RNAscope, ultrasensitive RNA *in situ* hybridization for *HTLV-1 bZIP factor* using RNAscope; HTLV-1, human T-cell leukemia virus type I.

Supplementary Figure 4. Correlation between PVL values in raw and FFPE tissue samples.

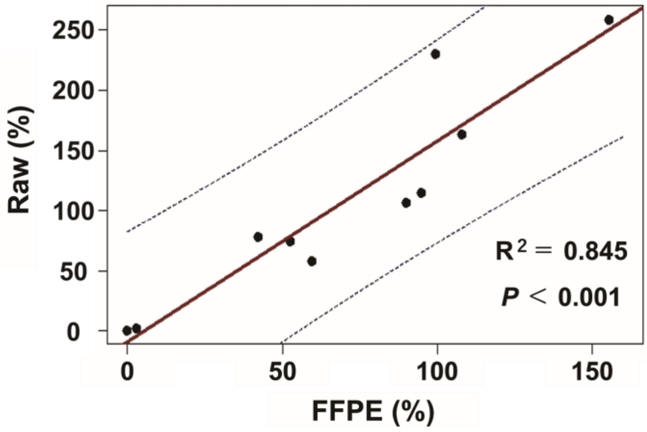

Correlations between PVL values in 10 FFPE and matched raw tissue samples were examined by simple regression analysis. The coefficient of determination (R^2) of 0.845 and *P* value < 0.001 indicated a high degree of correlation.

PVL, proviral load; FFPE, formalin-fixed paraffin-embedded.

Supplementary Figure 5. Unstable PVL value at low *HBB* copy number.

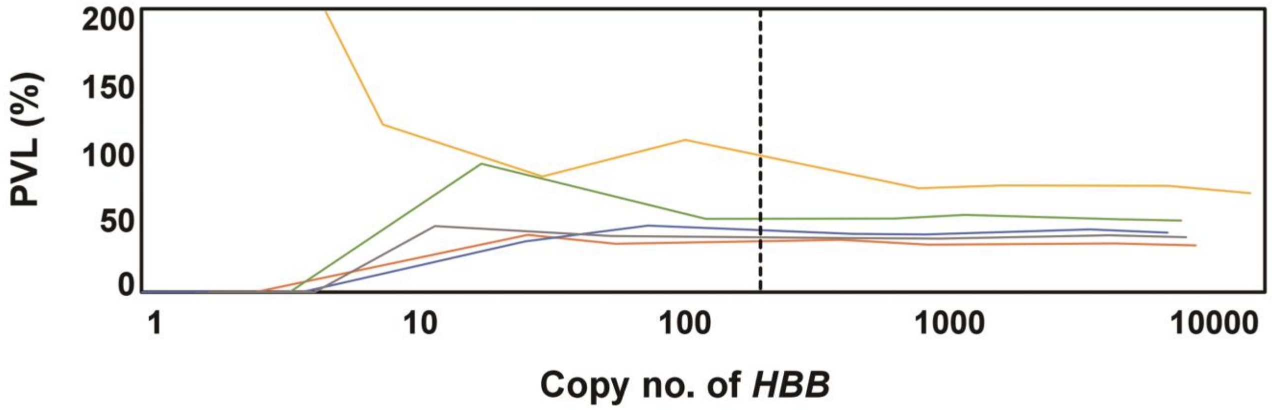

HTLV-1 quantification (*tax*-qPCR) was performed for five representative FFPE tissue samples with 7 serial dilutions of template DNA in order to assess the accuracy of the PVL value in cases with a low amount of input DNA. The measurement was performed three times simultaneously with the original sample and six dilutions for each specimen. The PVL value fluctuated markedly for *HBB* copy numbers ≤ 100. Therefore, only specimens with > 200 copies of *HBB* (indicated by the broken line) were deemed evaluable by *tax*-qPCR.

PVL, proviral load; HTLV-1, human T-cell leukemia virus type I; *tax*-qPCR, quantitative PCR detection of the *tax* gene; FFPE, formalin-fixed paraffin-embedded.

Supplementary Figure 6. An ATLL case in which monoclonal integration of HTLV-1 provirus was negative by SBH.

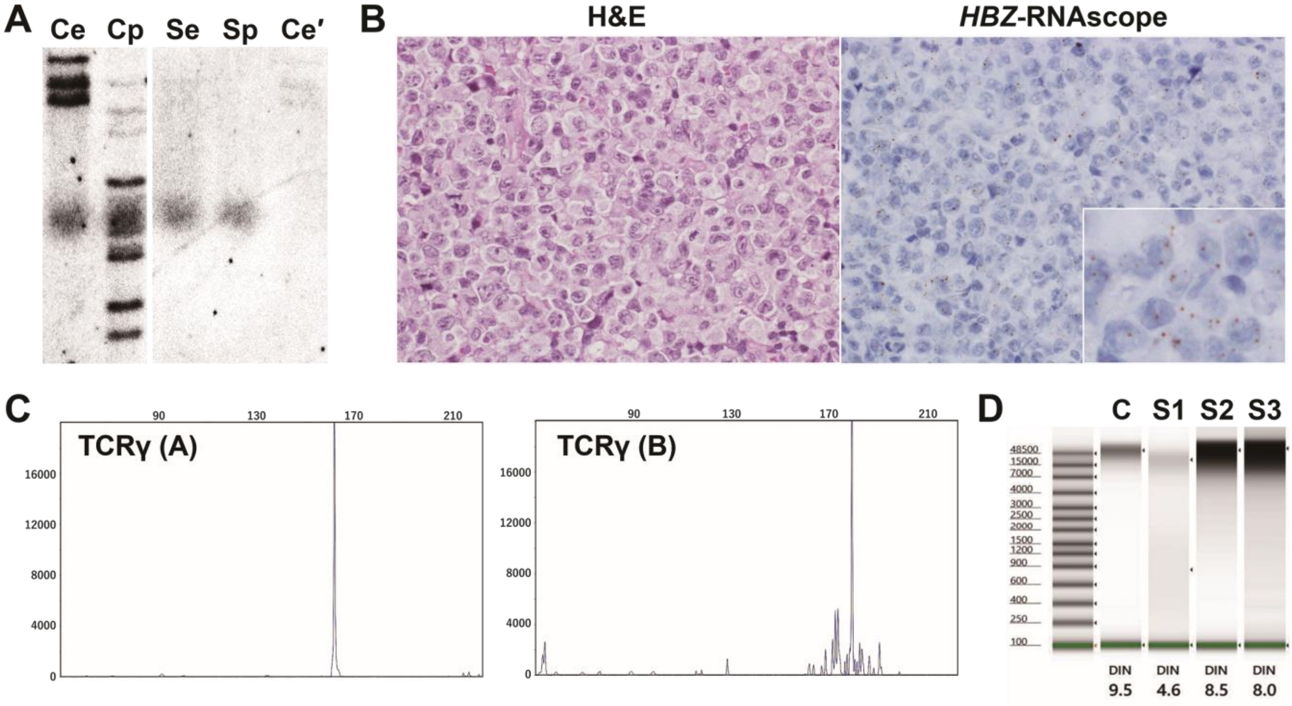

(A) SBH result using a fresh lymph node tissue sample at relapse, corresponding to the case no. 28. *Eco*RI-digested DNA (Se) and *Pst*I-digested DNA (Sp) showed no distinct bands related to HTLV-1 provirus, in contrast to the ATLL cell line ST-1 (Ce and Cp) and detection sensitivity control (*Eco*RI-digested DNA containing 4% ST-1 cells; Ce'). (B) H&E staining of the corresponding FFPE tissue sample revealed diffuse infiltration of atypical lymphocytes. *HBZ*-RNAscope confirmed *HBZ* signals in most of the cells in this area (scored as 1+). (C) PCR analysis of T-cell receptor (TCR) gene rearrangement according to the BIOMED-2 protocol performed with the DNA used for SBH. Clonality was detected in both the A and B tubes of TCR(γ) (evaluation range, horizontal axis; A: 145-255 bp, B: 80-140 bp, and 160-220 bp). (D) Quality of the DNA used in SBH analysis (S1) evaluated using TapeStation. The DNA integrity number (DIN) of S1 was 4.6, which was extremely low compared to sample C derived from a cell line (DIN = 9.5) and samples S2 and S3 derived from fresh tissue of ATLL patients (DIN = 8.5 and 8.0, respectively). S1, S2, and S3 were stored for similar lengths of time. Original magnification, 400× (B).

ATLL, adult T-cell leukemia/lymphoma; HTLV-1, human T-cell leukemia virus type I; SBH, southern blot hybridization; H&E, hematoxylin and eosin; *HBZ*-RNAscope, ultrasensitive RNA *in situ* hybridization for *HTLV-1 bZIP factor* using RNAscope; FFPE, formalin-fixed paraffin-embedded.

Supplementary Figure 7. *HBZ*-RNAscope result for non-ATLL lymphoid tumor arising in an HTLV-1 carrier.

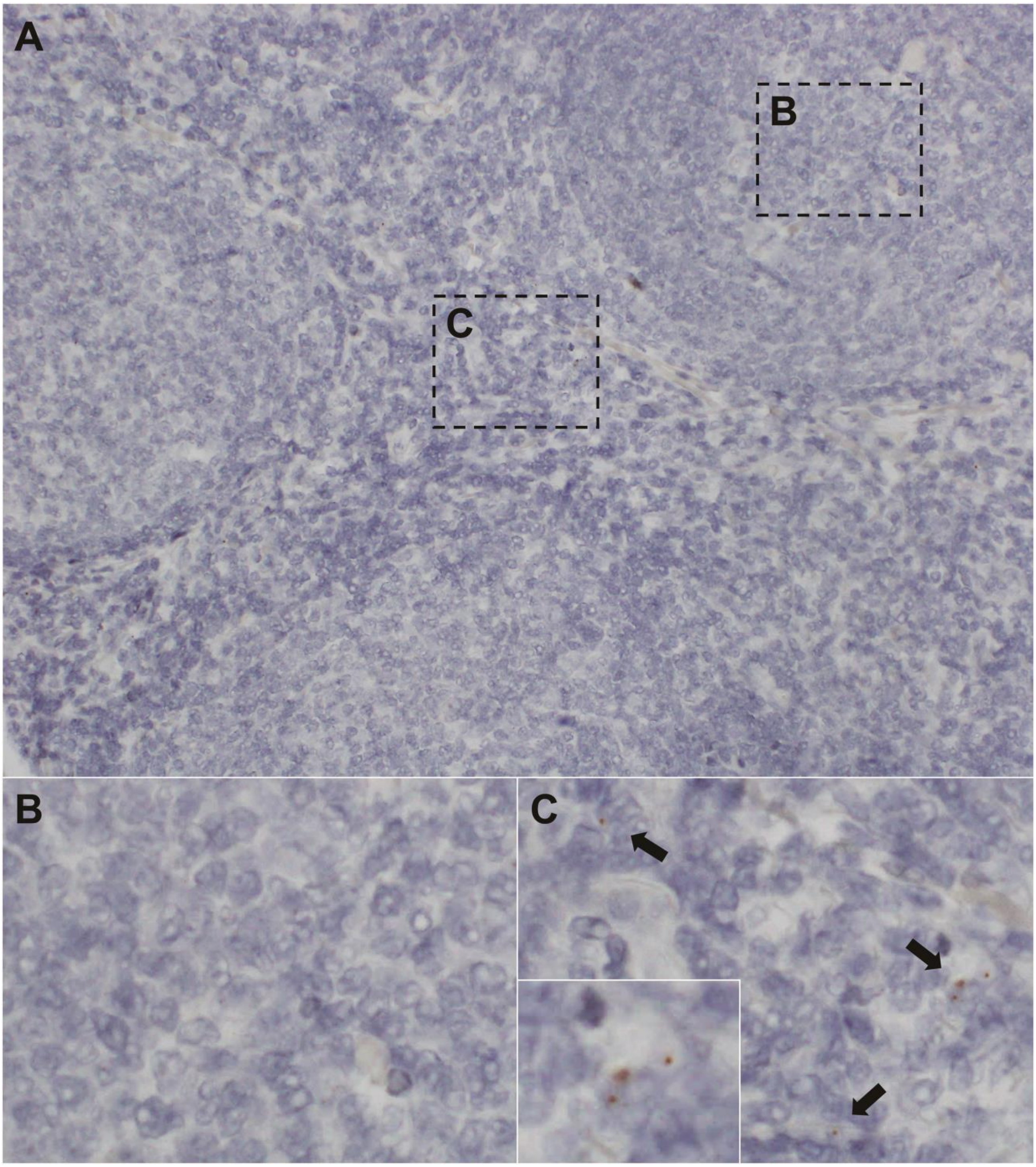

(A-C). *HBZ* signals. (A) *HBZ*-RNAscope of follicular lymphoma in an HTLV-1 carrier (case no. 74). Indistinct nodular (follicular) structures were observed. (B) No *HBZ* signal was observed in tumor cells located in follicular structures. (C) A small number of *HBZ* signals were detected in some cells located in the interfollicular area. Original magnification, 40× (A), 400× (B, C).

HBZ-RNAscope, ultrasensitive RNA *in situ* hybridization for *HTLV-1 bZIP factor* using RNAscope; ATLL, adult T-cell leukemia/lymphoma; HTLV-1, human T-cell leukemia virus type I.

Supplementary Figure 8. Visualization of HTLV-1-infected cells in a cutaneous lesion of ATLL.

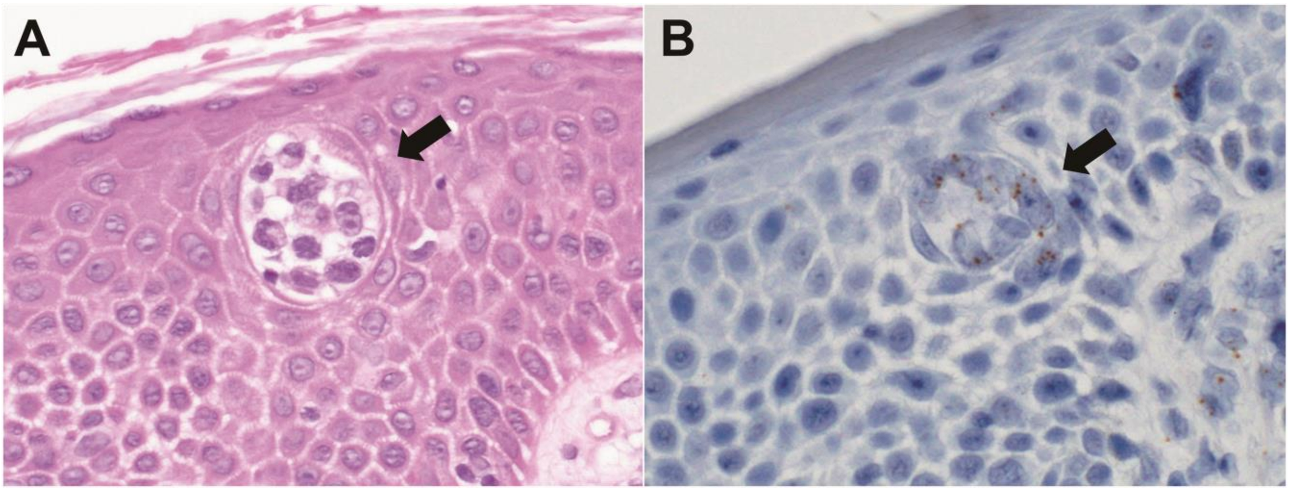

(A) H&E staining detected a Pautrier microabscess (case no. 120) as a cell aggregation with large atypical nuclei in the epidermis of the case diagnosed as an infiltration of ATLL. (B) *HBZ*-RNAscope revealed that the atypical cells were *HBZ*-positive, whereas no signals were observed in surrounding epidermal cells. Original magnification, 400× (A, B).

ATLL, adult T-cell leukemia/lymphoma; H&E, hematoxylin and eosin; *HBZ*-RNAscope, ultrasensitive RNA *in situ* hybridization for *HTLV-1 bZIP factor* using RNAscope.
